# Continuous multi‐day tracking of post‐myocardial infarction recovery of cardiac electrical stability and autonomic tone using electrocardiogram patch monitors

**DOI:** 10.1111/anec.13035

**Published:** 2023-01-11

**Authors:** Richard L. Verrier, Niraj Varma, Bruce D. Nearing

**Affiliations:** ^1^ Department of Medicine, Division of Cardiovascular Medicine, Beth Israel Deaconess Medical Center, Harvard Medical School Boston Massachusetts USA; ^2^ Cleveland Clinic Foundation Cleveland Ohio USA

**Keywords:** heart rate variability, non‐ST‐elevation myocardial infarction, ST‐elevation myocardial infarction, T‐wave alternans, T‐wave heterogeneity

## Abstract

**Background:**

Sudden cardiac death (SCD) risk is elevated following acute myocardial infarction (MI). The time course of SCD susceptibility post‐MI requires further investigation.

**Methods:**

In this observational cohort study, we employed state‐of‐the‐art noninvasive ECG techniques to track the daily time course of cardiac electrical instability and autonomic function following ST‐segment elevation myocardial infarction (STEMI) and non‐STEMI (NSTEMI). Preventice BodyGuardian MINI‐EL Holters continuously recorded ECGs for 7 days at hospital discharge and at 40 days for STEMI (*N* = 5) or at 90 days for NSTEMI patients (*N* = 5). Cardiac electrical instability was assessed by T‐wave alternans (TWA) and T‐wave heterogeneity (TWH); autonomic tone was determined by rMSSD‐heart rate variability (HRV).

**Results:**

TWA was severely elevated (≥60 μV) in STEMI patients (80 ± 10.3 μV) at discharge and throughout the first recording period but declined by 50% to 40 ± 2.3 μV (*p* = .03) by Day 40 and remained in the normal range (<47 μV). TWH, a related phenomenon analyzed from 12‐lead ECGs, was reduced by 63% in the five STEMI patients from discharge to normal (<80 μV) at follow‐up (105 ± 27.3 to 39 ± 3.3 μV, *p* < .04) but increased by 65% in a STEMI case (89 to 147 μV), who received a wearable defibrillator vest and later implantable cardioverter defibrillator. In NSTEMI patients, TWA was borderline abnormal (47 ± 3.3 μV) at discharge and declined by 19% to normal (38 ± 1.2 μV) by Day 90 (*p* = .05). An overall reciprocal increase in rMSSD‐HRV suggested recovery of vagal tone.

**Conclusions:**

This study provides proof‐of‐principle for tracking post‐MI SCD risk in individual patients with implications for personalized therapy.

## Graphical abstract

Microvolt T‐wave alternans (TWA) in a representative acute ST‐elevation myocardial infarction (STEMI) patient at hospital discharge. QRS‐aligned templates show that TWA was severely abnormal at 79 μV, indicating elevated arrhythmia risk.

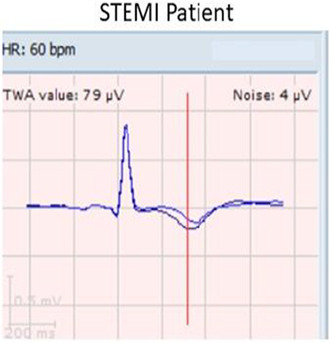

## 1 INTRODUCTION

Although the risk for sudden cardiac death (SCD) is known to be elevated following an acute myocardial infarction (MI), the precise extent and time course of changes in risk for lethal arrhythmias have not been adequately studied. Important insights were provided by Solomon et al. (2005), who conducted a retrospective analysis of the 14,609‐patient database from the Valsartan In Acute Myocardial Infarction Trial (VALIANT). The main finding was that after an MI, in individuals with left ventricular dysfunction, heart failure, or both, the incidence of SCD was highest in the first week after MI, fell rapidly in the first month, declined to one‐sixth at 1 year, and reached a nadir over the 3.5‐year follow‐up period. These observations suggest a critical role for early short‐term intervention. However, to implement this therapeutic strategy, it is essential to develop techniques for tracking risk on an individual patient basis, especially during the early weeks following an acute MI.

To address this challenge, we applied contemporary noninvasive methods. The first was high‐resolution multi‐day ambulatory electrocardiogram (AECG) patches capable of continuous monitoring across the critical weeks following ST‐elevation MI (STEMI) or non‐ST‐elevation MI (NSTEMI). The second approach involved quantitative assessment of T‐wave alternans (TWA) using the modified moving average (MMA) technique. This analytical method was cleared by the US Food and Drug Administration (FDA) and has been widely used and shown to be capable of tracking risk for life‐threatening arrhythmias during daily activities (Sakaki et al., 2009; Verrier et al., 2003; Verrier & Ikeda, 2013). The spectral method for TWA analysis was not employed because it requires fixation of heart rate and is not suitable for 24‐h AECG monitoring (Verrier et al., 2011). MMA‐based TWA has been shown capable of predicting intraprocedural percutaneous coronary intervention (PCI)‐related ventricular tachyarrhythmias (Takasugi et al., 2011; Verrier & Ikeda, 2013) and elevated TWA during the 2‐week period following MI has been shown to predict SCD (Hoshida et al., 2013; Verrier et al., 2003). T‐wave heterogeneity (TWH), a closely related electrophysiologic phenomenon and SCD predictor that can be measured on a 12‐lead ECG, was also determined. TWA (Verrier et al., 2011; Verrier & Ikeda, 2013) and TWH (Kenttä et al., 2016; Verrier & Huikuri, 2017; Verrier et al., 2021) have both been proven useful in predicting SCD, with significant hazard ratios of 4.2–22.6 and 2.8 (95% CI: 1.8–4.5), respectively. Since autonomic factors have been shown to play a major role in triggering arrhythmias post‐MI (Huikuri et al., 2001; La Rovere et al., 1998; Verrier & Antzelevitch, 2004), we also measured heart rate variability (HRV) using square root of the mean of the sum of squares of differences between adjacent normal RR intervals (rMSSD).

Our main hypothesis was that structural remodeling of the myocardial substrate and early recovery of cardiac eletrical stability and autonomic tone will be reflected in quantitative improvements in TWA, TWH, and rMSSD‐HRV, respectively, in individual patients as they recover from acute MI. We also postulated that patients with STEMI would exhibit higher initial levels of cardiac electrical instability compared with NSTEMI patients because of the greater severity of the coronary event.

## 2 METHODS

### 2.1 Standard protocol approvals, registrations, and patient consents

This observational cohort study conformed to requirements of the Declaration of Helsinki and the protocol was approved by the institutional review board of Beth Israel Deaconess Medical Center (BIDMC). The patients gave written informed consent prior to observational study procedures between January 2021 and December 2021.

### 2.2 Patient selection

Patients admitted to BIDMC for STEMI or NSTEMI requiring PCI were invited to participate. Inclusion criteria were as follows: ≥18 years old; recruited within 72 h after STEMI or NSTEMI; and required PCI. Exclusion criteria were as follows: (1) current device therapy or indication for device therapy [pacemaker, implantable cardioverter defibrillator (ICD) or cardiac resynchronization therapy device]; (2) permanent atrial fibrillation; (3) inability or unwillingness to give consent. An additional STEMI patient consented to participate but did not undergo discharge or follow‐up AECG monitoring because he received a wearable defibrillator vest at hospital discharge.

### 2.3 ECG recordings and analysis

**FIGURE 1:**
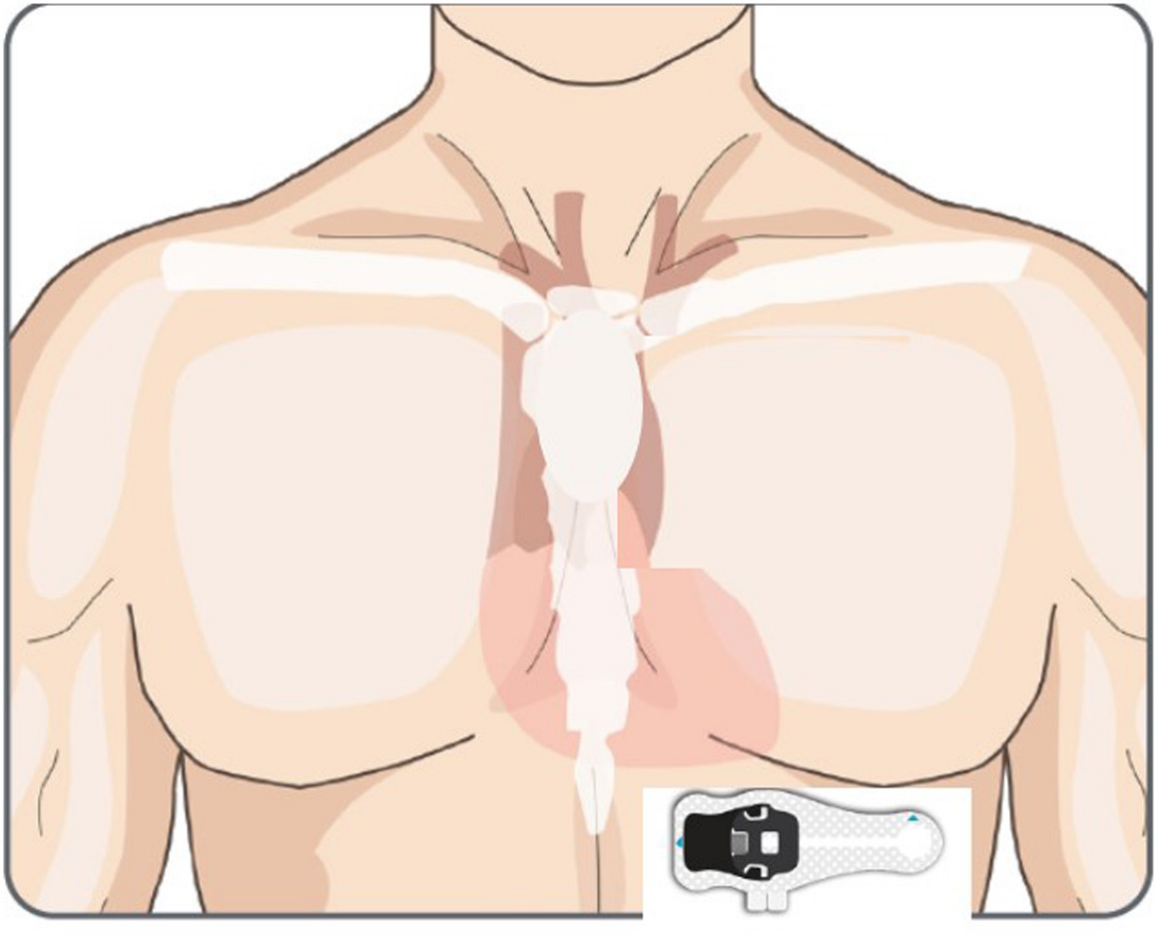
Illustration of the Preventice BodyGuardian MINI‐EL Holter and its placement on the torso in the V_4_, V_5_, and V_6_ regions to optimize T‐wave alternans detection (Verrier et al., 2011)

Consented, enrolled patients wore the Preventice BodyGuardian MiniHolter EL for 7 consecutive days post‐discharge and for 7 days at 40‐day follow‐up for STEMI patients (*N* = 5) or for 7 days at 90‐day follow‐up for NSTEMI patients (*N* = 5). The periods chosen are the standard‐of‐care recommendation for hospital visits to reassess left ventricular ejection fraction (LVEF) and medical therapy. When the consented patients were ready to be discharged from BIDMC, the Preventice BodyGuardian MiniHolter EL short strip was placed horizontally below the left pectoral muscle to record leads V_4_, V_5_, or V_6_ (Figure 1).

Analyses of TWA to assess cardiac electrical instability and of rMSSD‐HRV to determine autonomic function were performed using GE Healthcare's CardioDay ambulatory electrocardiogram diagnostic software by an investigator blinded to patients' clinical status (B.D.N.). Abnormal (≥47 μV) and severely abnormal (≥60 μV) TWA determinations (Verrier et al., 2011), abnormal TWH levels (>80 μV) (Kenttä et al., 2016; Tan et al., 2017), and depressed rMSSD‐HRV (<27 ± 12 ms) (Task Force, 1996) indicated “high risk” status. Analyses of TWH were performed from standard 12‐lead ECG recordings at rest with proprietary software (Nearing & Verrier, 2003) in the STEMI patients and the case.

### 2.4 Determination of TWA by modified moving average analysis of AECGs

This method was selected as it offers both practical and technical advantages. Specifically, TWA can be assessed from AECG recordings and does not require fixation of heart rate by either pacing or exercise. Technically, the method has proven capacity to quantify TWA as a continuous rather than binary (absent/present) variable. Briefly, the approach employs recursive averaging by dividing a stream of beats into odd and even bins and averaging the morphology of the beats in each bin over a few beats successively to create a moving average complex. TWA is computed as the maximum difference in amplitude between the odd‐beat and the even‐beat average complexes from the J point to the end of the T wave. TWA is averaged across 15‐s periods. The approach achieves an excellent signal‐to‐noise ratio, is relatively tolerant of nonstationary data such as changing heart rates or motion artifact, and is independent of phase‐shift perturbations (Nearing & Verrier, 2002). In the commercial FDA‐cleared software, algorithms have been incorporated to decrease the influence of noise and artifacts such as those caused by respiration. Quality control of automatically generated TWA values is achieved by beat labeling and exclusion of the suspect and preceding beats based on noise and prematurity according to several criteria, namely beats with >20 μV of noise measured during the isoelectric segments, regions with >25% of noisy beats, and ventricular premature beats (Verrier et al., 2011).

### 2.5 Determination of TWH by second central moment analysis from 12‐lead ECGs

This analytical approach was used to calculate TWH from 12‐lead ECG recordings at rest, as described in detail (Nearing & Verrier, 2003; Verrier et al., 2021; Verrier & Huikuri, 2017). Basically, the approach superimposes simultaneous waveforms from three precordial leads, typically V_4_, V_5_, and V_6_, and calculates the variance or splay about the mean waveform. A greater splay indicates a greater degree of nonuniformity of repolarization and correlates with increased risk for life‐threatening ventricular arrhythmias (Kenttä et al., 2016; Tan et al., 2017).

### 2.6 Determination of rMSSD‐HRV

HRV was determined using rMSSD, a time‐domain variable, because it is statistically robust and is able to detect labile changes in autonomic function (Task Force, 1996). RMSSD‐HRV is calculated as the square root of the squares of successive differences between RR intervals. Levels were computed for each patient in 5‐min segments at the time of the patient's daily maximum TWA level.

### 2.7 Statistics

Statistical analyses were performed using standard SAS software (SAS Institute, Cary, NC, USA). Time course of maximum TWA, TWH, and HRV was subjected to analysis of variance (ANOVA) with Tukey test for multiple comparisons. Discrete patient characteristics were analyzed with Fisher's exact test or log‐transformed variables if distribution was non‐normal. Values are reported as means ± SEM. Results were considered statistically significant when the *p*‐value was *<*.05.

## 3 RESULTS

### 3.1 Patient characteristics

The patient characteristics at baseline are provided in Table 1. Of the 10 patients studied, five had STEMI and five had NSTEMI, and seven were male. Characteristics of the groups were overall not significantly different, except for LVEF, which was lower in STEMI patients (46 ± 4%) compared with NSTEMI patients (61 ± 4%) (*p* = .024). None of the patients had heart failure. All patients underwent PCI as the first line of therapy with no residual stenosis following the procedure. The culprit arteries were variable. Only one STEMI patient experienced nonsustained ventricular tachycardia during the recorded follow‐up. One STEMI patient had a prior MI, and a second STEMI patient had previous coronary artery bypass graft surgery. At admission, two STEMI patients were receiving beta‐blockade therapy and none was receiving calcium channel blockade therapy or other antiarrhythmic medications. QRS duration (<150 ms) and QTc interval (<450 ms in men, <460 ms in women) (Cohagan & Brandis, 2022) were in the normal ranges for both STEMI and NSTEMI patients.

**TABLE 1:** Patient characteristics

	STEMI (*n* = 5)	NSTEMI (*n* = 5)
Mean age at diagnosis (years)	53.8 ± 6.5	58.8 ± 5.1
Male	5 (100%)	2 (40%)
PCI as first‐line therapy	5 (100%)	5 (100%)
Residual stenosis following PCI	0	0
Culprit artery	LAD (*n* = 1); LCx (*n* = 2); RCA (*n* = 1); LAD + LCx (*n* = 1)	LAD (*n* = 3); RCA (*n* = 1); LCx (*n* = 1)
NSVT	1 (20%)	0
Past or current smoker	2 (40%)	2 (40%)
Coronary artery disease	5 (100%)	5 (100%)
Heart failure	0	0
Previous myocardial infarction	1 (20%)	0
Previous CABG surgery	1 (20%)	0
Elevated troponin before PCI	3 (60%)^*^	5 (100%)
Elevated creatinine/creatine kinase before PCI	2	2
Beta‐blockers at admission	2 (40%)	0
Calcium channel blockers at admission	0	0
Other antiarrhythmic medications at admission	0	0
LVEF (%) in hospital	46 ± 4	61 ± 4
QRS (ms) at admission	94 ± 2	98 ± 5
QRS (ms) at discharge	97 ± 2	91 ± 2
QTc (ms) at admission	436 ± 15	438 ± 18
QTc (ms) at discharge	406 ± 10	451 ± 12

Abbreviations: CABG, coronary artery bypass graft; LAD, left anterior descending; LCx, left circumflex; LVEF, left ventricular ejection fraction; NSTEMI, non‐ST‐segment elevation myocardial infarction; NSVT, nonsustained ventricular tachycardia; PCI, percutaneous coronary intervention; RCA, right coronary artery; STEMI, ST‐segment elevation myocardial infarction.

^*^ The remaining 2 of 5 STEMI patients presented with chest pain and ST‐segment elevation. Diagnostic angiography confirmed coronary artery stenosis.

A sixth STEMI patient was consented and enrolled in the study, but his ECG patch monitor was discontinued to allow a wearable defibrillator vest to be placed. The case was a 54‐year‐old African American man weighing 187 lbs with a body mass index of 28.4 (i.e., overweight) and no history of smoking. After admission to the Emergency Department of BIDMC for chest pain, he was referred to the Cardiovascular Medicine Division, where he underwent echocardiography, which indicated reduced global left ventricular function consistent with extensive left anterior descending territory STEMI. The patient was diagnosed with ischemic cardiomyopathy due to acute systolic heart failure with an LVEF of 28%. The ECG patch monitor was placed on the day before hospital discharge but was removed after 4 h of recording so that he could receive a wearable defibrillator vest (Zoll Medical Corporation, Chelmsford, MA). However, it remained feasible to examine interlead TWH from the 12‐lead ECG recorded shortly before hospital discharge and at the 40‐day follow‐up visit.

### 3.2 T‐Wave Alternans and T‐Wave Heterogeneity

**FIGURE 2:**
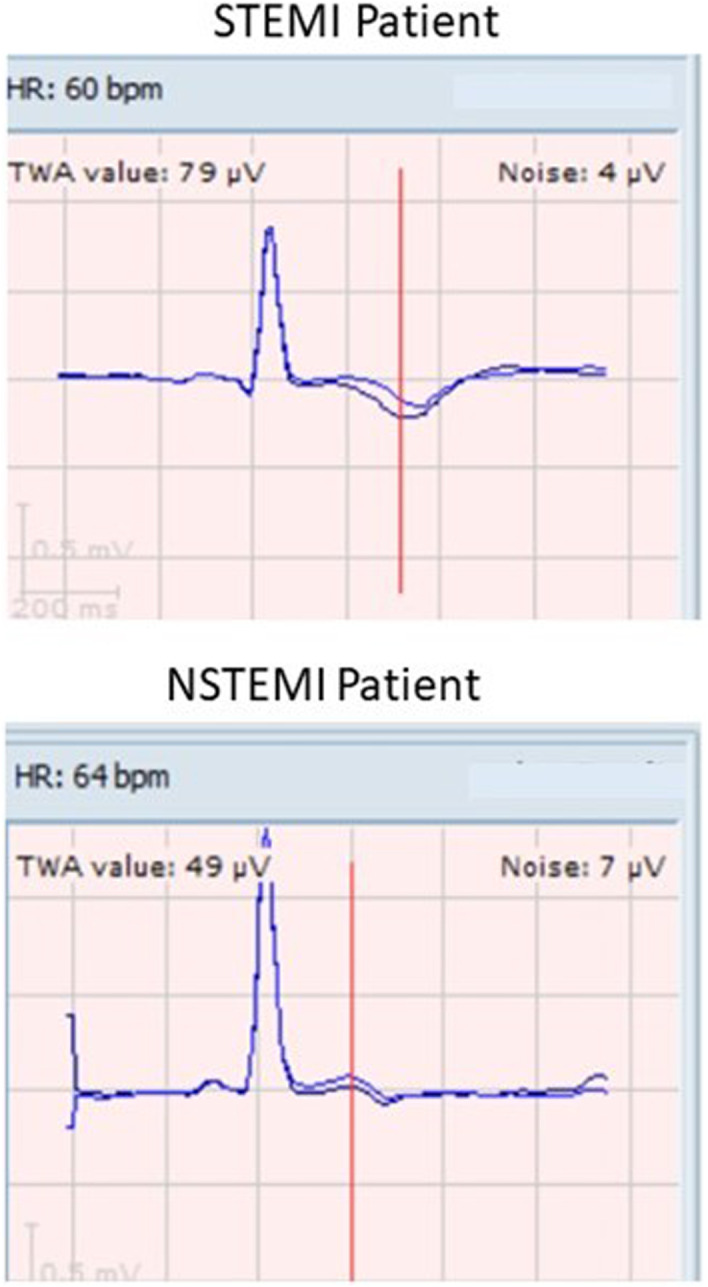
QRS‐aligned templates showing T‐wave alternans (TWA) in representative ST‐elevation myocardial infarction (STEMI) (upper panel) and non‐ST‐elevation myocardial infarction (NSTEMI) patients (lower panel) on the first day after hospital discharge. In the STEMI patient, TWA was 79 μV, which was severely abnormal (≥60 μV). In the NSTEMI patient, TWA was 49 μV, which is above the cutpoint of abnormality (≥47 μV) but was 38% lower than in the STEMI patient

**FIGURE 3:**
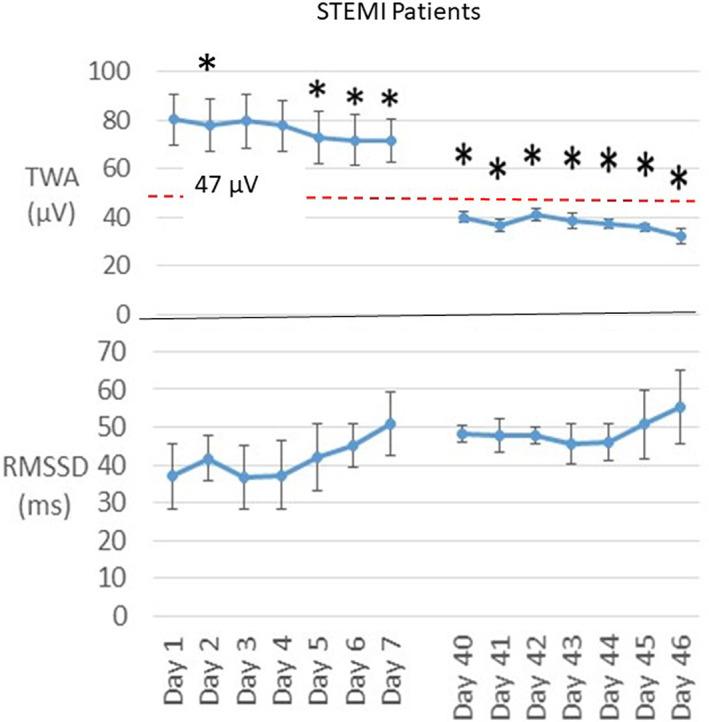
Time course of changes in T‐wave alternans (TWA) and heart rate variability (HRV) by analysis of square root of the mean of the sum of squares of differences between adjacent normal RR intervals (rMSSD) in five ST‐elevation myocardial infarction (STEMI) patients (group data). Upper panel: TWA was severely abnormal (≥60 μV) during the first 7 days after discharge but declined significantly to the normal range (<47 μV) by day 40, indicating a lowered level of risk for cardiac arrhythmias, and remained in the normal range during the second recording session. Lower panel: rMSSD‐HRV trended toward an increase during both recording periods. *p < .05 compared with discharge day

Reliable TWA data were obtained in all patients. Representative tracings for STEMI (upper panel) and NSTEMI patients (lower panel) are shown in Figure 2. Mean TWA was markedly elevated on hospital discharge in five STEMI patients to 80 ± 10.3 μV (Figure 3, upper panel), well above the ≥60 μV TWA cutpoint of severe abnormality. Recovery of TWA in STEMI patients was prolonged, as it did not decline to normal levels (<47 μV) within the initial 7‐day recording period. Although TWA decreased significantly on the first day after discharge, it remained above the ≥60 μV cutpoint of severe abnormality. By the 40th day after discharge, TWA had declined by 50% to 40 ± 2.3 μV (*p* = .03), with all patients in the normal range, and continued in the normal range throughout the second recording period.

**FIGURE 4:**
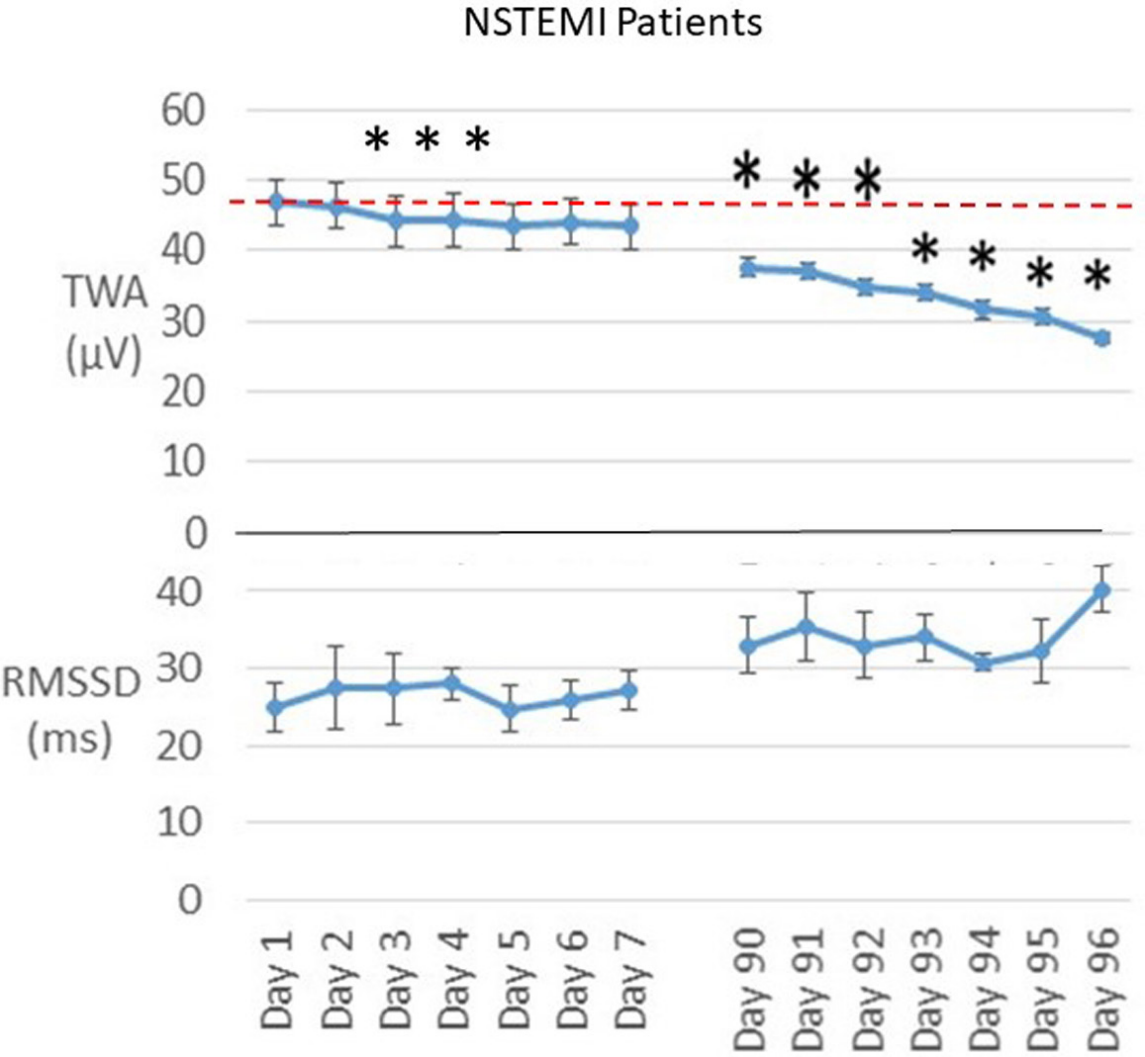
Time course of changes in T‐wave alternans (TWA) and heart rate variability (HRV) by analysis of square root of the mean of the sum of squares of differences between adjacent normal RR intervals (rMSSD) in five non‐ST‐elevation myocardial infarction (NSTEMI) patients (group data). Upper panel: TWA was in the borderline abnormal range (~47 μV) during the first recording period and in the normal range (<47 μV) during the second recording period, indicating a lowered level of risk for cardiac arrhythmias. Lower panel: A reciprocal increase in rMSSD‐HRV was prominent during the second follow‐up visit during a significant decline in TWA. *p < .05 compared with discharge day

Patients with NSTEMI exhibited TWA in the borderline abnormal range at hospital discharge (47 ± 3.3 μV) that declined to 44 ± 3.7 μV on Day 3 (*p* = .005) (Figure 4, top panel). By the 90th day after hospital discharge at the start of the second recording period, TWA had dropped by 19% to 38 ± 1.2 μV (*p* = .05), when all NSTEMI patients exhibited TWA in the normal range.

**FIGURE 5:**
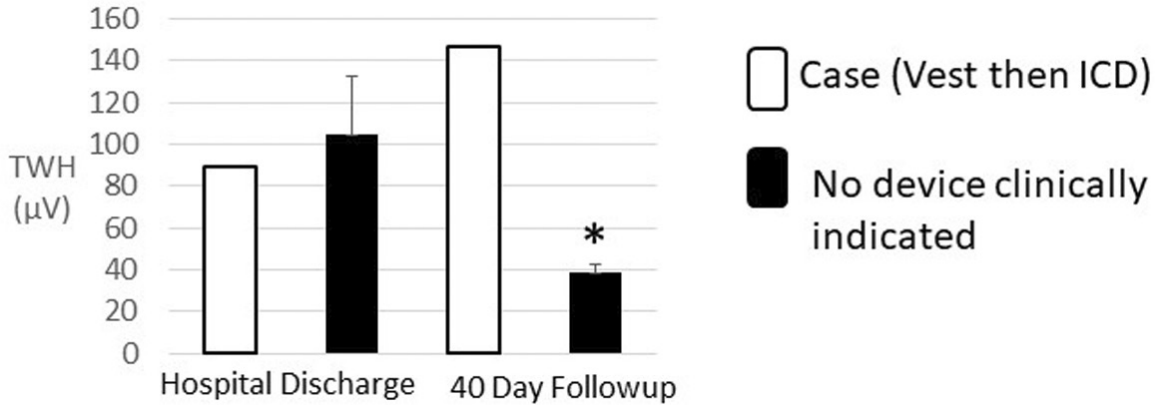
Pattern of recovery of T‐wave heterogeneity (TWH) in the ST‐elevation myocardial infarction (STEMI) case who required a wearable defibrillator vest and implantable cardioverter defibrillator (ICD) (open bar) compared with STEMI patients (*n* = 5) without clinical indications for a vest or ICD (filled bar). At hospital discharge, TWH was abnormal (>80 μV) at 89 μV in the STEMI case. At the 40‐day follow‐up, TWH had increased by 65% to 147 μV. By comparison, in the five STEMI patients without indications for a vest or ICD, TWH was elevated (105 ± 27.3 μV) at hospital discharge. In the three patients with 12‐lead ECGs at the 40‐day follow‐up, TWH decreased by 63% to normal levels (39 ± 3.3 μV, *p < .04). Data are reported as means ± SEM

TWH levels at hospital discharge and at follow‐up in the STEMI case and in the five STEMI patients who did not require a vest or device were examined (Figure 5). At hospital discharge, the case had severely elevated TWA (79 μV) and TWH (89 μV). At the 40‐day follow‐up, TWH had increased by 65% to 147 μV. Shortly after ICD placement, the patient experienced an appropriate defibrillator discharge. The five STEMI patients who did not require a device exhibited a 63% decrease in TWH level from hospital discharge to 40‐day follow‐up (105 ± 27.3 to 39 ± 3.3 μV, *p* < .04).

### 3.3 Heart rate variability

The time course of rMSSD‐HRV showed an overall reciprocal increase suggesting recovery of cardiac vagal tone in STEMI and NSTEMI patients. In the five patients who experienced STEMI, there was a trend toward progressive improvement in rMSSD‐HRV (Figure 3, lower panel). On the follow‐up visit at 40 days post‐discharge, rMSSD‐HRV showed a continued trend toward an improvement at 46 days. This pattern appeared to show an improvement in cardiac vagal tone that was reciprocal to the reduction in TWA. In the NSTEMI patients, the main changes in rMSSD‐HRV appeared to be delayed, accompanied by a corresponding improvement in TWA in the second monitoring period (Figure 4, lower panel).

### 3.4 QTc interval

QTc intervals were normal in the STEMI and NSTEMI patients at hospital discharge and follow‐up. In the case, QTc interval was normal (438 ms) at discharge and slightly elevated (462 ms) at the 40‐day follow‐up.

### 3.5 Heart rate

**FIGURE 6:**
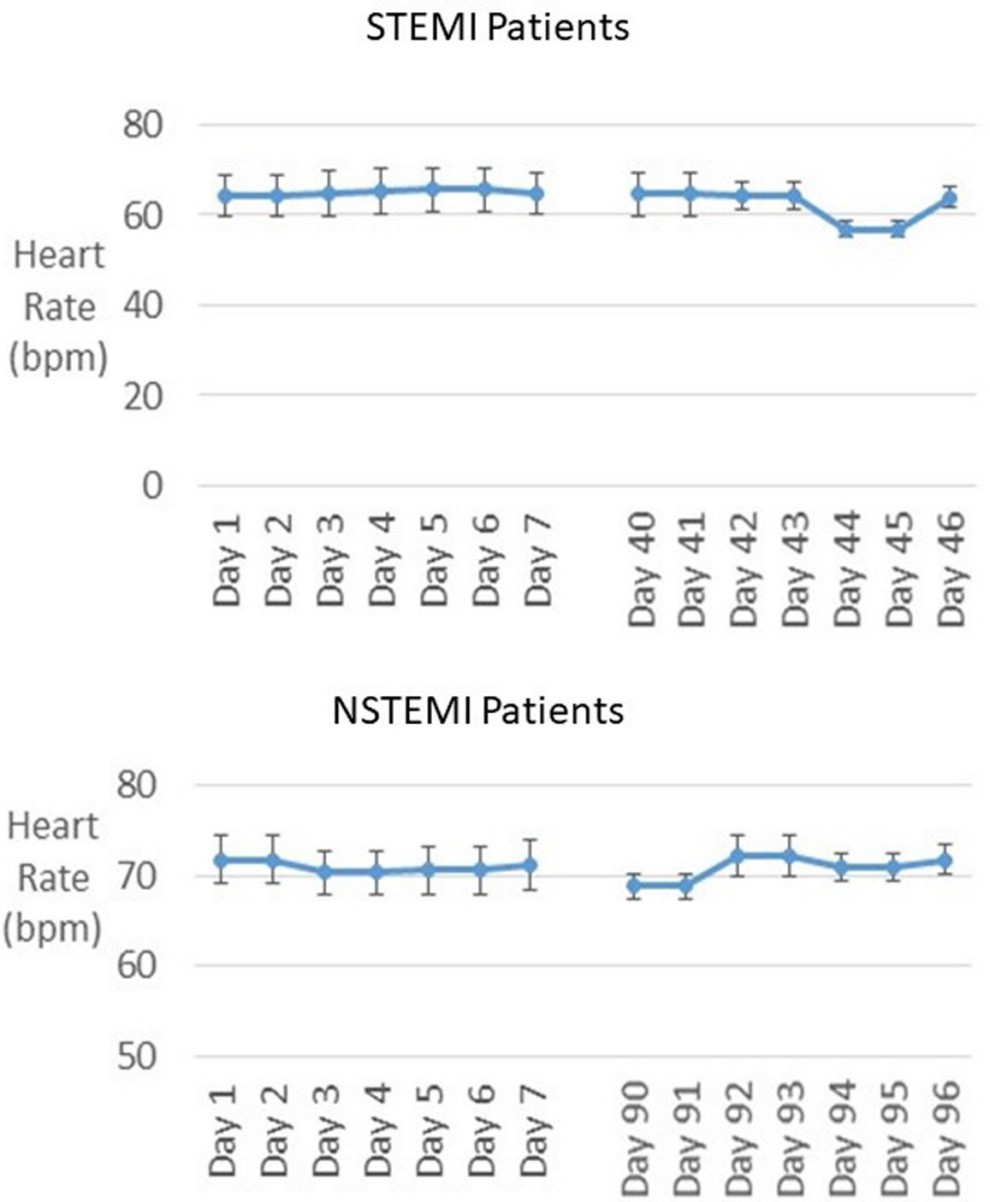
Time course of heart rate in ST‐elevation myocardial infarction (STEMI) (upper panel) and non‐ST‐elevation myocardial infarction (NSTEMI) patients (lower panel). Heart rate was remarkably stable from day to day in both groups, likely due to the fact that all patients were receiving beta‐blockade therapy

Heart rate was remarkably stable from day to day in both STEMI and NSTEMI groups (Figure 6), likely due to the fact that all patients were receiving beta‐blockade therapy.

## 4 DISCUSSION

### 4.1 Main findings

This is the first study to demonstrate that cardiac electrical instability, as assessed by TWA, is markedly elevated on hospital discharge in STEMI patients. A progressive recovery in TWA in STEMI patients was evident at Days 5, 6, and 7, followed by a decrease to the normal range by the start of the scheduled STEMI follow‐up visit at Day 40 (Figure 3). Patients with NSTEMI showed a more gradual recovery in TWA, which was in the borderline abnormal range during the first session and in the normal range during the second monitoring period (Figure 4). The time course of TWA recovery was matched by an apparent reciprocal increase in cardiac vagal tone as assessed by rMSSD‐HRV. The STEMI case who required a wearable defibrillator vest and ICD exhibited increased TWH at follow‐up (Figure 5).

### 4.2 Prior studies

Analysis of TWA magnitude as a marker of risk for cardiac arrest or SCD on AECG recordings was initially examined in patients enrolled in the Autonomic Tone and Reflexes after Myocardial Infarction (ATRAMI) Study (Verrier et al., 2003). TWA levels were measured from 24‐h AECGs obtained approximately 2 weeks post‐MI. Elevated TWA ≥47 μV was associated with 4‐ to 7‐fold odds of cardiac arrest or arrhythmic death during the 21 ± 8‐month follow‐up. Hoshida et al. (2013) confirmed these overall findings and reported in a sizeable study of >300 post‐MI patients that elevated TWA was associated with a hazard ratio of 5.8 for fatal arrhythmic events including SCD. High levels of TWA also predicted the occurrence of nonsustained ventricular tachycardia during PCI in post‐MI patients (Takasugi et al., 2011; Verrier et al., 2013). In the Finnish Health Survey 2000, TWH analyzed from resting 12‐lead ECGs identified subjects who died suddenly with 2.8 (1.8‐4.5) hazard ratios (*p* < .001) (Kenttä et al., 2016; Verrier & Huikuri, 2017; Verrier et al., 2021). The results of the ATRAMI study also demonstrated the predictive capacity of altered autonomic tone (HRV) and baroreceptor reflex sensitivity (BRS) in post‐MI patients (La Rovere et al., 1998). Specifically, both impaired HRV and depressed BRS measured in the same 24‐h period predicted arrhythmic death on an individual patient basis during the nearly two‐year follow‐up. While the long‐term stability of rMSSD‐HRV following MI has been previously reported (Task Force, 1996), daily levels in the immediate post‐MI period have not been previously investigated.

### 4.3 Current study

The present study was motivated by the milestone investigation of Solomon et al. (2005), who demonstrated in their retrospective analysis of the VALIANT study that incidence of SCD was highest in the first week after MI, decreased rapidly during the first month, and reached a nadir over a 3.5‐year period. The authors reported that their results indicated a critical role for early short‐term intervention to avert cardiac events during the high‐risk early period. However, to pursue this therapeutic strategy, techniques would need to be developed for tracking risk on an individual patient basis.

Our study was designed with this specific challenge in mind. We postulated that the use of multi‐week AECG patch monitoring with analysis of TWA and rMSSD‐HRV would permit tracking of structural remodeling of the myocardial substrate and changes in autonomic tone.

Our findings demonstrate a correspondence between the time courses of recovery of SCD risk and the reduction in TWA levels and improvement in rMSSD‐HRV following MI. In the five patients with STEMI, an established high‐risk population, TWA was severely abnormal (≥60 μV) at ~80 μV for the first 4 days post‐discharge and was reduced but remained severely abnormal for the next 3 days (Figure 3). This pattern concurs with that reported by Solomon et al. (2005) of marked risk for SCD during the first week following discharge. Thereafter, TWA decreased to the normal range (<47 μV) by 40–47 days of follow‐up. The pattern of recovery of TWA in NSTEMI patients was comparable with that observed in STEMI patients although the initial TWA level at hospital discharge in NSTEMI patients remained borderline abnormal (~47 μV) during the first 7 days post‐discharge (Figure 5). TWH exhibited a 63% decrease from hospital discharge to follow‐up (Figure 5). The single exception was the STEMI case whose TWH level increased 65% from discharge to follow‐up.

In the five STEMI patients, the decline in TWA during the first week post‐MI was accompanied by a trend toward a reciprocal increase in rMSSD‐HRV (Figure 3). This finding suggests an increase in cardiac vagal tone. As vagus nerve stimulation has been shown to reduce TWA both experimentally (Verrier et al., 2009) and clinically (Nearing et al., 2021; Schomer et al., 2014), it is plausible that recovery of vagal influences may have been in part responsible for the progressive decline in TWA observed during post‐MI recovery. This mechanism may also be operative in NSTEMI patients. The recovery pattern of rMSSD‐HRV differed somewhat from STEMI patients as the larger effect was evident during the clinically scheduled follow‐up visit at 90 days post‐discharge (Figure 4). The TWA decrease was correspondingly greatest during this timeframe.

The basis for the time course of recovery is unclear. One possibility based on the medical literature is that baroreceptor sensitivity, another indicator of baroreceptor function that can influence cardiac vagal tone, improves progressively during post‐MI recovery (La Rovere et al., 1998).

Neither QRS duration nor QTc interval reflected known elevated arrhythmia risk. Current evidence indicates that QTc prolongation is a relatively poor predictor of SCD risk in the general population [hazard ratio = 1.5 (95% CI: 1.2–1.8), *p* < .0002] as compared to TWA and TWH (Porthan et al., 2013; Verrier & Huikuri, 2017).

### 4.4 Limitations

The main limitation of the current study is its relatively small sample size. However, it should be noted that the determinations of TWA and rMSSD‐HRV are based on nearly 7000 h of recordings. The relatively small variability in the data as shown in the standard errors of the means lends further support to the robustness of the results. A second limitation is that the sample size was not sufficient to observe outcomes such as cardiac arrest or malignant arrhythmias. Thus, our inferences are based on the correspondence of the TWA and rMSSD‐HRV changes with the findings of prior outcome investigations including the VALIANT (Solomon et al., 2005) and ATRAMI studies (Verrier et al., 2003). In the future, it will be valuable to extend continuous AECG patch‐based monitoring to 30 or more days during recovery of cardiac electrical stability and autonomic function, which exhibit a critical time‐sensitive progression following STEMI and NSTEMI. Such long‐term monitoring could enable improved tracking of ongoing cardiac substrate remodeling and “neural rewiring” of the heart (Verrier & Kwaku, 2004; Zhou et al., 2004).

## 5 CONCLUSIONS AND IMPLICATIONS

This hypothesis‐generating proof‐of‐principle study suggests that by utilizing current clinically available noninvasive tools including AECG patches and measurement of TWA and rMSSD‐HRV; the time course of change in arrhythmia risk can be tracked in individual patients on a daily basis during acute post‐MI recovery. The results are encouraging as the changes match the SCD risk pattern disclosed by prior large studies (Solomon et al., 2005). Collectively, these observations demonstrate the value of concurrent monitoring of indices of cardiac electrical instability and autonomic tone during myocardial substrate and neural remodeling (Hoshida et al., 2013; Verrier & Antzelevitch, 2004). The fact that MMA‐based TWA analysis has been shown specifically to reflect risk for fatal arrhythmic events including SCD (Hoshida et al., 2013; Verrier et al., 2003) is relevant to considering this marker and analytical approach for patient selection for ICD placement (Verrier et al., 2011).

A potential direct application of this approach is suggested by a national study of adherence to beta‐blockade therapy following MI (Kramer et al., 2006). Remarkably, drug adherence fell by 31% within the high‐risk first 30 days after discharge. As TWA is highly responsive to adrenergic influences, an upsurge in TWA could be a signal of noncompliance to beta‐blockade therapy by individual patients and an increase in attendant risk for life‐threatening arrhythmias. Since beta‐blockade therapy is standard of care, the use of AECG patch monitoring of TWA to track adherence could constitute a significant future application.

Overall, in terms of potential impact on clinical practice, ECG patch‐based TWA and 12‐lead ECG‐based TWH are capable of detecting elevated levels of cardiac electrical instability in individual post‐MI patients. These indices also have the potential to track the time course of changes in cardiac electrical instability during post‐MI remodeling and could prove helpful in monitoring the propensity for life‐threatening arrhythmias following STEMI. TWA and TWH assessment could improve arrhythmia risk tracking over standard QTc interval measurement. Finally, as some ECG patches have telemetry capabilities, including certain models of the monitors employed in the current study, daily or on‐demand interrogation by caregivers is feasible.

## AUTHOR CONTRIBUTION

Dr. Verrier interpreted the data and drafted the manuscript. Dr. Varma critically reviewed the manuscript. Dr. Nearing analyzed and interpreted the data.

## CONFLICT OF INTEREST

None of the authors has a conflict of interest. Drs. Verrier and Varma are members of the Editorial Board of Annals of Noninvasive Electrocardiology. To avoid bias, they were excluded from all editorial decision‐making related to the acceptance of this article for publication.

## ETHICAL APPROVAL

This observational cohort study conformed to requirements of the Declaration of Helsinki, and the protocol was approved by the institutional review board of Beth Israel Deaconess Medical Center. The patients gave written informed consent prior to observational study procedures between January 2021 and December 2021.

## Data Availability

The data are not publicly available.
